# Case of Recovery from an Abdominal Abscess

**Published:** 1834-01-01

**Authors:** John Valentine

**Affiliations:** Somerton, Somersetshire


*To the Editor of the Medical Quarterly Review.*

Sir: The following brief account of a case of recovery from an abdominal abscess, after parturition, may not be uninteresting to your readers. In the works which I have an opportunity of consulting, I find but very little said on the subject, excepting in Howship's "Practical Observations in Surgery and Morbid Anatomy;" with the statement of the dissection of one of whose cases I shall close this paper, and, by thus placing it by the side of mine, it may serve to elucidate the nature of this formidable disease.

Your obedient servant,

John Valentine, m.r.c.s.l.

*Somerton, Somersetshire; December 18th,* 1833.

"Inflammation of the peritoneum is a frequent occurrence, and in almost every case a very serious one. Affections of this nature sometimes run on very quickly to purulent effusion; and, when this happens, I believe, there are exceeding few, if any, well-authenticated instances of recovery. Should the inflammation, however, fall rather short of the above effect, so as to connect itself with the pouring out of coagulable lymph only, even in this case the event must be extremely precarious, particularly when the effusion has taken place to any considerable degree."—*Howship's Practical Observ. in Surgery*, page 228.

Case. Ann Sheppard, æt. twenty-one, was delivered, the 5th of December, 1832, of her first child. She was in labour twelve hours. A full secretion of milk took place at the usual period, and she continued well for a fortnight after, excepting occasional pinching pains of the bowels, like labour-pains. At the end of this time she was attacked with pain of the head and delirium, frequent shiverings, fever, and increased pain of the abdomen, with great tenderness, diarrhœa, and suppression of the milk and lochia. The medical man who had attended the patient in her labour being sent for, prescribed saline medicines, and ordered the hair to be cut very short, and the head to be bathed with vinegar.

At the end of the fourth week I was requested to see her, and found her labouring under a violent cough and difficulty of breathing, to which she had been subject long before her confinement. The abdomen, which was much enlarged, was painful on pressure, and the fluctuation of a fluid could be distinctly perceived. There were frequent rigors, followed by fever, and the pulse was 120. I gave her medicines calculated to allay fever and irritation. In a few days the umbilicus began to rise, and became as large as a common-sized apple, red and shining. Poultices were applied, and in about a month the tumour burst, and discharged a gallon of pus, of a white colour, but soon turning green in the cloths, with shreds of membrane floating in it. Considerable quantities came from the orifice daily, and the discharge did not cease till the end of four months. The health then improved: the cough and difficulty of breathing completely subsided, and the patient is now better than she was before the illness occurred.

Mr. Howship having described a case, which, excepting in its termination, was very similar to the one just related, gives the following post-mortem appearances:

"The body was extremely emaciated. On passing a probe by the opening at the umbilicus, a small sinus was traced, leading obliquely underneath the integuments, and through the muscles, towards the pubes, to the extent of three inches, where it found an open way into the general cavity of the abdomen.

"On laying open the cavity of the abdomen, the probe was ascertained to have entered by a small opening ulcerated through the peritoneum. The peritoneum covering the anterior parietes of the abdomen had suffered considerable inflammation, and about two pints of purulent matter, with some flocculent masses of coagulable lymph, were removed from the abdomen and pelvis.

"There was no mark of disease about the uterus, or its appendages."—*Howship*, page 231.

Smellie gives a case which he entitles "A Case of a violent Inflammation of the Uterus, an imposthume forming, and discharged at the navel." The discharge continued several weeks, by which the patient was much weakened; but at last she recovered.

